# Evaluating Overhead Sprinklers and Sprayers for Heatwave Protection in Avocado Orchards

**DOI:** 10.3390/plants15101516

**Published:** 2026-05-15

**Authors:** Arnon Dag, Helena Vitoshkin, Guy Resef, Yonatan Ron, Victor Alchanatis

**Affiliations:** 1 Gilat Research Center, Agricultural Research Organization, Volcani Institute, M.P. Negev, Gilat 8528000, Israel; yron@agri.gov.il; 2 Institute of Agricultural Engineering, Agricultural Research Organization, Volcani Institute, Rishon LeTsiyon 7505101, Israel; elenav@agri.gov.il (H.V.); victor@volcani.agri.gov.il (V.A.); 3 Netafim Orbia, Hatzerim 5320047, Israel; guy.reshef@netafim.orbia.com

**Keywords:** avocado, climate change, evaporative cooling, water stress, heat stress, salinity

## Abstract

With global climate change, heatwaves have become more frequent and severe in avocado-growing regions. High temperatures combined with wind and low humidity are problematic for avocados, especially during the early developmental stage of the young fruitlets. Hence, heatwaves during this phenological stage are considered a major limiting factor for avocado productivity. This study evaluated the effects of operating pulsing sprinklers or sprayers installed above the canopy during spring heatwaves over three consecutive seasons in a Hass avocado orchard. We evaluated foliage and fruitlet temperature (using remote and proximal sensing), stem water potential, stomatal conductance, salt accumulation on the leaves, and productivity. The cooling system reduced the foliage temperature by 6–8 °C and fruitlet temperature by 5–10 °C with respect to uncooled trees. Stem water potential was increased by 0.8–2.0 MPa in the treatment plots compared to the control. The cooling treatments led to an average 42% yield increase over the next 3 years. No significant differences were found between the sprinklers and sprayer for any of the measured parameters. Results indicate the effectiveness of an evaporative cooling system for mitigating heatwave damage and improving avocado productivity.

## 1. Introduction

The avocado (*Persea americana*), native to Mexico, Central America, and northern South America, has emerged as a premier subtropical fruit with increasing global demand, primarily driven by its recognized nutritional profile and documented human health benefits [1]. Consequently, its global production increased by over 170% between 2009 and 2019, with international exports reaching approximately 2.5 million tons in 2021 [2]. While Mexico remains the leading producer (2.4 million tons), followed by Colombia (0.87 million tons) and the Dominican Republic (0.67 million tons), the global export market is dominated by a single cultivar—Hass—which accounts for roughly 90% of the trade [2,3].

The expansion of avocado cultivation into regions far from its center of origin has exposed the crop to sub-optimal environmental conditions [4]. ‘Hass’ trees thrive within a relatively narrow thermal window, with optimal temperatures ranging from 14 to 18 °C at night and from 18 to 25 °C during the day [5,6]. Temperatures exceeding 33 °C are considered a critical threshold for physiological damage, particularly during the sensitive phenological stages of pollination and fruit set [4,7]. In regions such as Israel, the primary climatic risk involves extreme fluctuations in temperature and humidity during the spring season [8]. Such heat stress interferes with vital physiological processes, including stomatal conductance, photosynthesis, respiration, and the regulation of leaf water potential [9,10,11]. As climate-change models predict an increase in the frequency, duration, and severity of compound drought and heatwave events [12], developing effective mitigation strategies is imperative.

Evaporative cooling systems have been found effective at reducing heat-stress damage in various crops, including grapes [13], apples [14,15,16,17], winter wheat [18], and strawberries [19]. The amounts of water applied can be small because such systems are not intended to wet the rooting zone but to increase evaporative cooling and reduce the vapor-pressure deficit (VPD) of the microclimate around the crops.

We previously demonstrated the efficacy of evaporative cooling systems in protecting avocados against heatwaves [20]. However, those systems utilized discharge rates of 1.7–3.1 mm/h, which can put a strain on the hydraulic capacity of pressurized irrigation systems during heatwaves when water demand is already at its peak. The current study evaluates the performance of low-discharge cooling devices (1.5 mm/h). Reducing the discharge rate is essential for commercial adoption, as it allows growers to maintain standard irrigation schedules while simultaneously providing canopy cooling. Hence, while the previous study established the general efficacy of evaporative cooling, this research moves from a proof of concept to commercial viability and physiological precision. Furthermore, while our previous work noted salt accumulation on leaves following canopy wetting, it did not distinguish between surface deposits and salts absorbed into the leaf tissue [20]. This study aims to quantify the proportion of salts penetrating the leaves by comparing the mineral content of washed and unwashed leaves, thereby clarifying the potential long-term impact on leaf functionality.

## 2. Results

### 2.1. Stem Water Potential (SWP)

**Figure 1:**
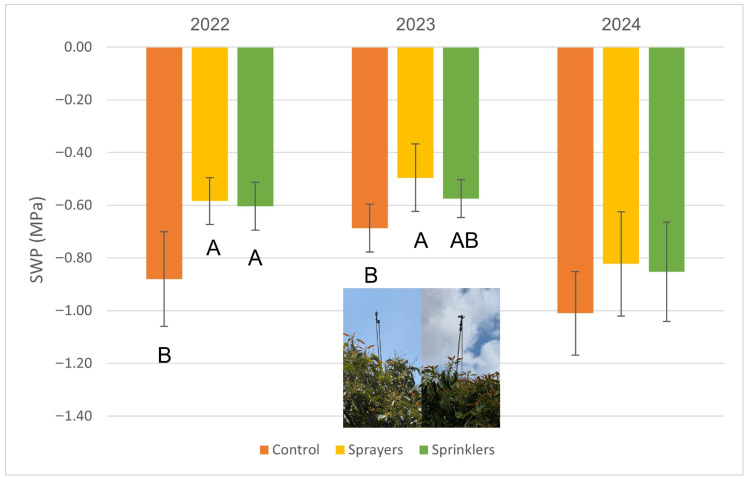
Stem water potential (SWP) of ‘Hass’ avocado trees during a heatwave, as affected by canopy-cooling treatment at Kibbutz Ruhama, 2022–2024. Different letters indicate significant differences between treatments (*p* < 0.05). Bars are standard deviation (SD) values.

Water stress, as reflected by SWP, was consistently about 0.2 MPa lower in the evaporative cooling treatments (Figure 1), with sprayers and sprinklers exhibiting similar levels of SWP reduction.

### 2.2. Stomatal Conductance

**Figure 2:**
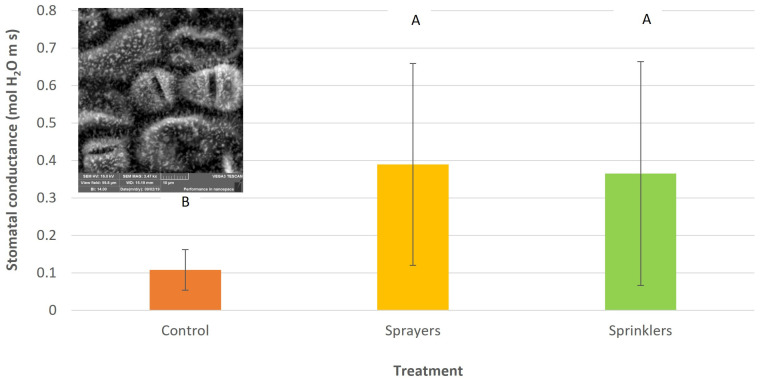
Stomatal conductance of ‘Hass’ avocado trees during a heatwave, as affected by canopy-cooling treatments at Kibbutz Ruhama, 9 May 2024. Different letters indicate significant differences between treatments (*p* < 0.05). Bars are SD values. Upper-left corner: electron micrograph of 3 stomata on an avocado leaf (photo by S. Lazare).

Stomatal conductance increased significantly—more than 3-fold—following operation of the evaporative cooling system (Figure 2). The two cooling treatments (sprayers and sprinklers) had similar effects on stomatal conductance.

### 2.3. Yield

**Figure 3:**
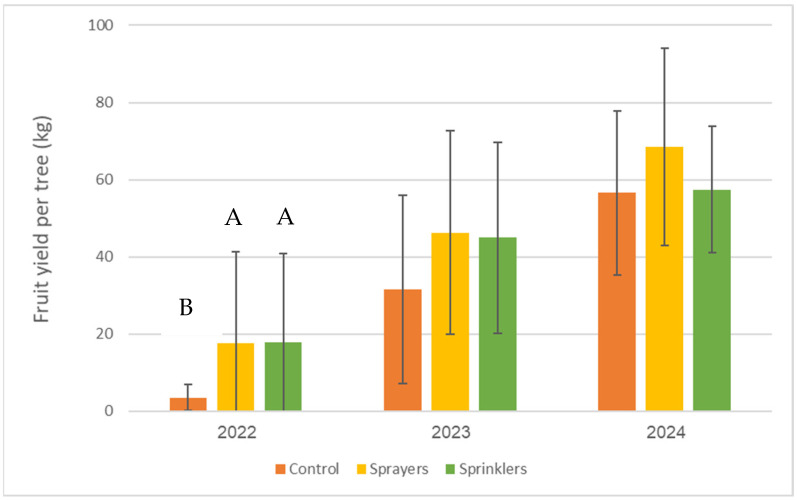
Yield in ‘Hass’ avocado trees, as affected by canopy-cooling treatments at Kibbutz Ruhama, 2022–2024. Different letters indicate significant differences between treatments (*p* < 0.05). Bars are SD values.

Figure 3 shows the effects of evaporative cooling treatments on annual fruit yield. The yield in the treated trees was consistently higher than that in the controls for all 3 years. However, the effect was more pronounced in 2022 and 2023 and significant only in 2022. The average yield was significantly higher (*p* < 0.05) for the cooling treatments compared to controls: 30.6 kg for the control, 44.2 kg for the sprayer treatment, and 40.1 kg for the sprinkler treatment.

### 2.4. Leaf Salt Accumulation

Calcium (Ca) did not appear to accumulate in the leaves following evaporative cooling (Table 1). Surprisingly, in both years, the potassium (K) level was lower in leaves exposed to the sprayer treatment. In 2023, chlorine (Cl) levels doubled in leaves treated with the evaporative cooling system, with slightly lower values for sprinklers compared to sprayers. No such effect was observed in 2024, probably due to the system’s shorter operation time (Table S1). Washing the leaves barely reduced their Cl levels, indicating that most of it was absorbed by the plant; a similar trend was observed for sodium (Na), i.e., a significant increase following the evaporative cooling treatment and a slight reduction after washing.

**Table 1:** Mineral content (average ± SD) in washed and unwashed leaves.

Year	Washed Leaves	Mineral	Control	Sprayers	Sprinklers
2023	No	Ca	1.25 ± 0.26 A	1.16 ± 0.36 A	1.27 ± 0.31 A
	No	K	0.85 ± 0.17 A	0.65 ± 0.11 B	0.84 ± 0.23 A
	No	Na	0.01 ± 0.01 B	0.04 ± 0.01 A	0.04 ± 0.01 A
	No	Cl	0.1 ± 0.05 B	0.22 ± 0.04 A	0.20 ± 0.02 A
	Yes	Ca	1.03 ± 0.22 A	1.04 ± 0.27 B	1.04 ± 0.19 A
	Yes	K	0.82 ± 0.16 A	0.56 ± 0.16 B	0.61 ± 0.23 B
	Yes	Na	0.01 ± 0.003 B	0.03 ± 0.01 A	0.03 ± 0.01 A
	Yes	Cl	0.10 ± 0.03 C	0.21 ± 0.04 A	0.17 ± 0.06 B
2024	No	Ca	1.47 ± 0.29 A	1.42 ± 0.19 A	1.46 ± 0.25 A
	No	K	1.09 ± 0.20 A	0.88 ± 0.19 B	1.06 ± 0.22 A
	No	Na	0.02 ± 0.01 B	0.03 ± 0.01 A	0.03 ± 0.01 A
	No	Cl	0.24 ± 0.1 A	0.23 ± 0.10 A	0.23 ± 0.07 A
	Yes	Ca	1.24 ± 0.29 A	1.31 ± 0.33 A	1.10 ± 0.26 A
	Yes	K	0.97 ± 0.21 B	0.8 ± 0.19 A	0.96 ± 0.19 AB
	Yes	Na	0.01 ± 0.01 A	0.01 ± 0.01 A	0.02 ± 0.01 A
	Yes	Cl	0.21 ± 0.08 A	0.22 ± 0.06 A	0.22 ± 0.06 A

Different letters in a row indicate a significant difference between treatments (*p* < 0.05).

### 2.5. Canopy Temperature

**Figure 4:**
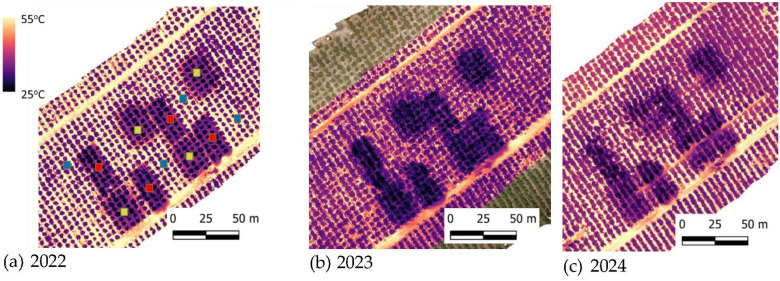
Canopy temperature maps of avocado trees at Kibbutz Ruhama acquired from a height of 50 m with an FLIR SC655 thermal camera mounted on a DJI M600 drone: (**a**) 2022, (**b**) 2023, and (**c**) 2024.

**Figure 5:**
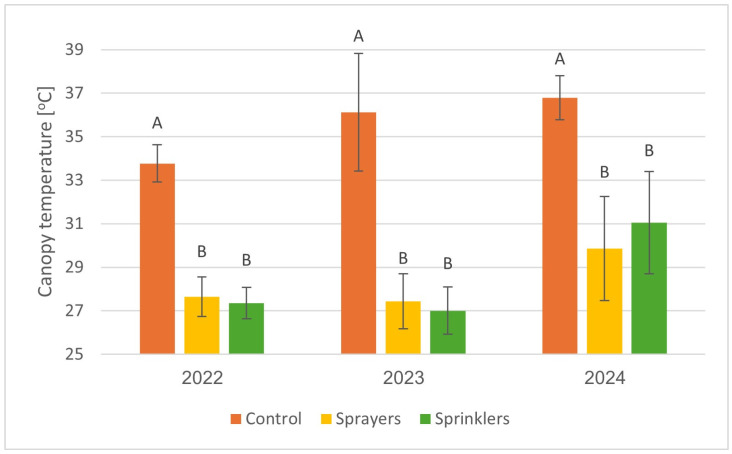
Average canopy temperature from UAV thermal imaging at Kibbutz Ruhama for 2022, 2023, and 2024. Different letters indicate significant differences between treatments (*p* < 0.05). Bars are SD values.

Canopy temperature and system efficacy were evaluated. Aerial thermal imaging, which was used to monitor canopy temperatures during heat events in 2022, 2023, and 2024, demonstrated significantly lower foliage temperatures for the cooling treatments vs. controls (Figure 4 and Figure 5). In the Ruhama orchard, the temperature gap between cooled and uncooled trees was approximately 6 °C in 2022 and 2024. This cooling efficiency was maintained and even slightly increased in 2023, where the recorded difference reached approximately 8 °C. Results from all years showed no significant difference in canopy-temperature reduction between trees cooled by sprayers and those cooled by sprinklers.

### 2.6. Fruitlet Temperature

**Figure 6:**
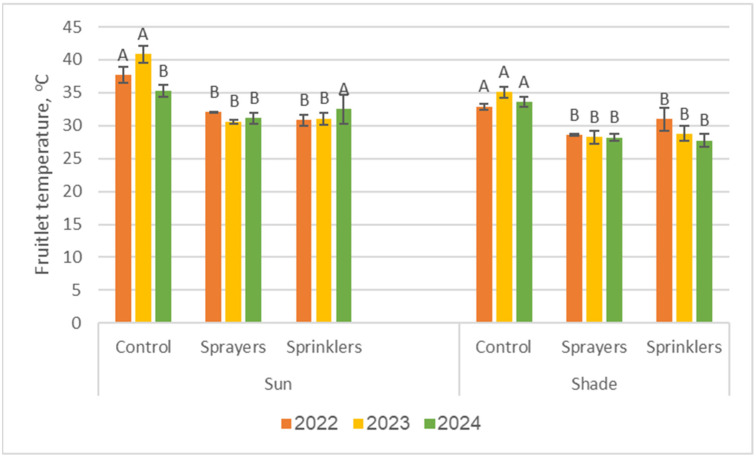
Mean fruitlet temperatures measured in the three experimental years (26 June 2022, 4 May 2023, and 24 April 2024) under control conditions and the two cooling treatments, comparing sun-exposed and shaded fruitlets. Measurements were taken using handheld thermal imaging of 9–10 fruitlets per tree (≈5 sun-exposed and ≈5 shaded). Different letters above the bars indicate significant differences among treatments within each canopy position and year (*p* < 0.05). Bars are SD values.

The results from the three measurement years (Figure 6) showed a consistent pattern of elevated fruitlet temperatures under heat-stress conditions, especially for sun-exposed fruitlets in the untreated control plot. In the second experimental year (measurement on 4 May 2023), which experienced the most severe weather conditions of the study, the mean fruitlet temperatures in the control plot reached 41 ± 0.3 °C in sun-exposed positions, a level considered abnormally high and potentially harmful. In shaded-canopy positions, temperatures remained below 35 °C, demonstrating the protective effect of the foliage.

Across all 3 years, clear and significant differences (*p* < 0.05) were observed between treatments. Under sun-exposed conditions in each year, the control treatment consistently showed significantly higher fruitlet temperatures compared to the cooled plots. Thus, across all 3 years, cooling treatments significantly lowered fruitlet temperatures, with differences of 5–10 °C relative to untreated fruitlets on hot days: sun-exposed fruitlets were cooled to approximately 30 °C, while shaded fruitlets were cooled to values below 30 °C. Consistent with previous findings, no substantial differences were observed between the two cooling systems. Under similar weather conditions, mean fruitlet temperatures were 29 ± 3 °C with sprayers and 26 ± 2 °C with sprinklers.

**Figure 7:**
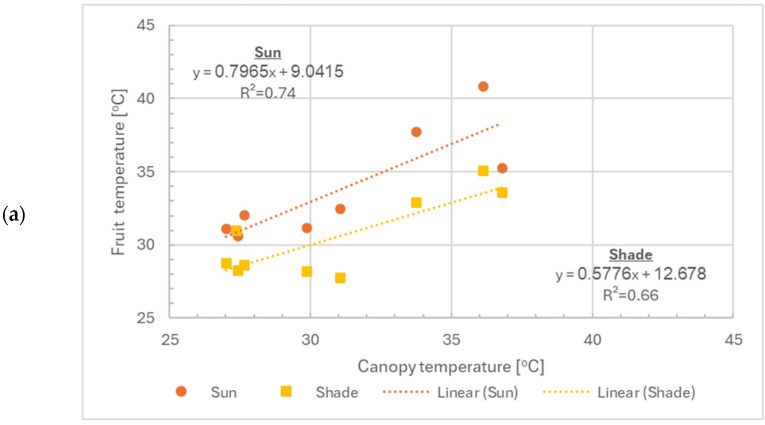

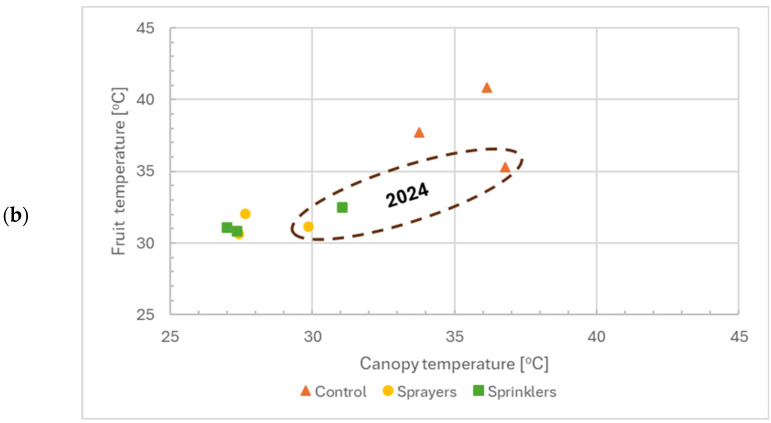
Relationship between canopy temperature and fruitlet temperature during the heat events of all trees: cooled and uncooled. (**a**) Fruitlets in the shade and fruitlets under direct sun radiation. (**b**) Fruitlets under direct sun radiation, depicting cooling treatments and year of measurement.

Canopy temperature was correlated with fruitlet temperature for both exposed and shaded fruitlets (Figure 7a), with R^2^ values of 0.74 and 0.66, respectively. This relationship was similar for 2022 and 2023 but slightly different for 2024 (Figure 7b). This difference might be attributed to differences in the meteorological conditions during the day of the measurement campaign or to differences in canopy structure (volume, etc.) across years.

## 3. Discussion

With global climate change, heatwaves are expected to become more frequent. Since the 1950s, the largest changes in climate extremes have occurred for daily temperature measurements—including heatwaves [21]—a trend that is expected to continue in the future [22]. The increasing frequency and severity of heatwaves present a critical challenge for avocado production. Avocado trees are particularly vulnerable during the early fruit-set stage, when high temperatures often lead to massive fruitlet abscission and subsequent yield loss.

While mitigation strategies such as shading nets can improve photosynthetic activity and productivity [10], they are often prohibitively expensive and labor-intensive due to their temporary use. Other methods, such as kaolin or calcium carbonate sprays, only offer limited efficacy [23,24]. Another solution is to select and breed new varieties and clones of cv. Hass which are more tolerant to heat stress [11]. However, our findings demonstrate that a low-discharge evaporative cooling system provides a superior, scalable solution by reducing tree water stress and heat stress and, subsequently, increasing yield; the more extreme the heat-event conditions, i.e., the hotter and drier the ambient air, the greater the cooling-system efficiency [8]. The cooling treatments consistently improved yield (Figure 3), but their effect was more pronounced in 2022. The differences among years might be a result of differences in heatwave severity (daily maximum temperature), duration, or timing relative to flowering and early fruit set each year—these three parameters were integrated in the ‘heat stress’ index for avocado trees in a model developed by Lomas [7]; the higher the ‘heat stress index’, the higher the relative contribution of the cooling system to yield.

The cooling system effectively reduced VPD by lowering the air temperature and increasing relative humidity [25]. This microclimatic shift explains the marked improvement in SWP observed in the treated plots (Figure 1), which likely prevented the accelerated fruitlet abscission typically triggered by water stress [26] and further enhanced stomatal opening (Figure 2) for continued carbohydrate assimilation. Hence, it seems that the improved stomatal conductance following evaporative cooling treatments is due to both the improvement in tree water stress and the direct effect of reduced VPD [27]. The plant-based water stress indicator SWP (Ψ_stem_) and canopy temperature are commonly considered in studies assessing water stress in avocados [28]. The SWP in the treatment plots reached levels of moderate stress [29], between −0.5 and −0.6 MPa in 2022 and 2023, whereas in 2024, the treatment presented higher stress levels (0.8 MPa), probably due to insufficient irrigation. In this respect, Schaffer et al., 2024 [27] reported that stomatal conductance begins to decline progressively at SWP levels of −0.4 MPa and continues until the stomata close completely at levels of −1.0 to −1.2 MPa, consequently decreasing photosynthesis. Those SWP values were found in 2022 and 2024 in the control plots.

Lomas and Mandel [8] reported a pronounced reduction of 7 °C in avocado plantations when sprinklers were operated during a heat event (when the temperature at 0800 h was 28 °C and above). However, they used a high discharge rate of 7 mm/h, whereas in the current study, we used only 21% of this amount (1.5 mm/h) for a significant reduction in temperature and an increase in avocado productivity. In our previous study, with a discharge rate of 1.7–3.1 mm/h, we observed a 10 °C reduction in the canopy temperature [20]. It seems that the reduced canopy temperature in the present study (Figure 4 and Figure 5) reflects the combined effect of evaporative cooling and improved water status of the avocado trees (Figure 1), which allowed stomatal opening (Figure 2) and leaf cooling due to enhanced evapotranspiration. The discharge rate is critical for adopting the cooling system in commercial orchards; during a heat event, growers generally irrigate their plantation, and the pipe’s hydraulic capacity for the delivery of additional water for cooling is limited.

High temperatures can increase fruitlet drop and might cause some reduction in fruit quality [30]. The mean fruitlet temperature in our study was 5–10 °C lower in treated trees compared to that in controls (Figure 6). In a previous study of apples in Washington state, fruit temperature was reduced by 3.1–5.8 °C following the use of an evaporating cooling system [31]. Unfortunately, there is almost no literature on young avocado fruitlet temperatures under high ambient temperatures and their effect on the developing fruit’s physiology and quality. However, we might expect an increase in sunburn incidence and internal damage in the mature fruit.

Salinity remains a major threat to avocado productivity worldwide, especially in Mediterranean regions where low-quality water is used for irrigation [32,33]. In general, salts are absorbed from the soil by the roots, but they can be absorbed by the leaf as well [34] when irrigating with sprinklers. The fact that leaf washing reduced the accumulation of salts (Na and Cl) only slightly indicates absorption by the leaf. Cl is generally considered the most harmful element in terms of salinity in avocado, with a threshold for damage of 0.25% [35]. This threshold was not exceeded in the current study. However, when using low-quality water for evaporative cooling in avocado, salinity damage might occur [20]. Still, the effect of salinity would probably be temporary, as opposed to the continuous effect incurred with brackish water irrigation, where Na accumulates in the trunk with time [36]. This is because of the limited time exposure to salinity and the tendency of leaves exposed to salinity to drop and be replaced by new leaves when using evaporative cooling.

Despite the differences in droplet size and operation intervals (continuous for sprayers vs. pulses for sprinklers), both treatments showed similar efficacy with respect to SWP, stomatal conductance, canopy and fruitlet temperatures, leaf salt accumulation, and yield. Hence, it seems that the pulse sprinklers are a better choice for the avocado grower because each sprinkler covers a radius of 7 m, whereas with the sprayer system, each tree requires one sprayer; the former can thus be installed to cover eight trees, making it more cost-effective from the perspectives of installation costs and maintenance. The average annual amount of water used for evaporative cooling was 40.5 mm, which is ca. 5% of the total amount of water used for orchard irrigation; hence the water costs involved in evaporative cooling are negligible.

## 4. Materials and Methods

### 4.1. Experimental Setup

The experiment was conducted in a ‘Hass’ avocado orchard located near Kibbutz Ruhama (31°29′17.1″ N 34°42′27.7″ E) in the Western Negev region of Israel. The soil was classified as Loess soil with 40–50% sand, 20–30% silt, and 25–35% clay. The experimental site has a typical Mediterranean climate, with hot and dry summers, mild winters, annual precipitation averaging ~330 mm and falling exclusively from October until April and concentrated over November–March, and an average relative humidity of 60–65%. The Ruhama avocado orchard is planted on an area of 24 ha. The experimental plot was planted on 2 ha at a density of 4.5 × 5 m between trees (440 trees/ha) and consisted of cv. Hass; every third tree in every third row was cv. Ettinger, which was planted as a pollinizer. Trees are pruned every year, with a height of approximately 3 m and a width of 3 m. The orchard is drip-irrigated using emitters with a flow rate of 1 L/h spaced every 0.3 m in one lateral line per tree row. Fresh water is used for irrigation and cooling, originating mainly from the desalinization plant (22 mg/L Cl, 23 mg/L N, 34 mg/L Ca, 1 mg/L K, and an electrical conductivity of 325 µS/cm). The thresholds for automated activation of the cooling system were set to air temperatures above 36 °C and relative humidity below 30%; detailed operation times are presented in Table S1. In general, the system was operated five times during 2022 (for a total of 20.4 h), seven times in 2023 (total: 35.0 h), and four times (days) in 2024 (total: 28.4 h). Two types of cooling systems with a similar discharge rate of 1.5 mm/h were tested: sprayers (SuperNet, Netafim, Israel) and pulse sprinklers (MegaNet, Netafim). Sprinklers and sprayers were located approximately 0.5 m above the tree canopy. Each treatment was tested in four replicates, with each replicate consisting of 8 trees in 3 adjacent rows (24 trees per replicate); the inner trees were used for measurements and the outer ones were used as ‘border’ trees.

### 4.2. Measurements

#### 4.2.1. SWP

On 26 June 2022 (1st season), 4 May 2023 (2nd season), and 9 May 2024 (3rd season), while the cooling system was active, two mature leaves from each of the four trees per plot were enclosed in aluminum bags for 2 h before SWP measurement. The measurement was conducted at noon using a Scholander-type pressure chamber (MRC, Holon, Israel).

#### 4.2.2. Leaf Analysis

Twenty healthy mature leaves from the canopy of two trees per plot were sampled on 14 June 2022 (1st season), 19 June 2023 (2nd season), and 3 July 2024 (3rd season) shortly after cessation of the cooling treatments. The leaves were dried at 70 °C in a well-ventilated oven. Each sample was then ground and thoroughly mixed. The quantity of Cl in the leaf was determined based on water extraction (0.1 g of dry matter in 10 mL of deionized water) using an MKII Sherwood M926 Chloride Analyzer, Sherwood Scientific, Cambridge, UK. Na and Ca were determined by digesting the powdered material with nitric acid and H_2_O_2_ and analyzing them in an ICP-OES 5100 apparatus (Agilent Technologies, Santa Clara, CA, USA). K concentrations in the leaf blades, petiole, and berry powder were determined by atomic absorption spectrophotometry (Perkin-Elmer model 460, Waltham, MA, USA) after digestion with sulfuric acid.

#### 4.2.3. Yield

Fruit were harvested at the time of commercial harvest in the orchard: 4 December 2022 (1st season), 3 December 2023 (2nd season), and 20 January 2025 (3rd season). Each tree was harvested individually, and its yield was weighed.

#### 4.2.4. Canopy Temperature

The tree canopy temperature was mapped during major heatwaves using an FLIR SC655 thermal infrared camera, Wilsonville, OR, USA mounted on a drone. The camera is radiometrically calibrated, based on an uncooled microbolometer focal array, with a sensitivity of 0.1 degrees and a radiometric accuracy of less than 2 degrees. The images were acquired from 50 m above ground level with a 24 mm lens, resulting in a ground spatial resolution of 3 cm/pixel. The flight plan included 70% overlap between adjacent legs and 90% overlap in the flight direction. Seven ground-control points were placed within the scanned area, and their geographical coordinates were measured using an RTK GPS (Spectra Precision SP60 L1/L2 GNSS receiver (Spectra Geospatial (part of Trimble), Westminster, CO, USA) with a handheld Trimble TDC600 interface, (Tipp City, OH, USA), with 1 cm accuracy. The produced map was georeferenced from the acquired images using Pix4d commercial software (Pix4Dmapper v4.7 and v4.8, Pix4D S.A., Lausanne, Switzerland).

In 2022, due to a limited number of natural heat events in the spring season, the cooling systems were manually activated on hot days (23 June) to test their cooling capacity. Thermal imaging was conducted simultaneously with the operation of the cooling treatments. In 2023, flights were conducted during a heat event in Ruhama on 4 May and again on 8 August. Data from the heat event on 4 May 2023 are presented in this paper. In 2024, a flight was conducted on 9 May.

#### 4.2.5. Fruitlet Temperature

Fruitlet temperatures were measured using handheld thermal imaging device (FLIR TG165 thermal camera, Wilsonville, OR, USA) during the critical fruitlet-development stage over three consecutive years (2022–2024). Thermal measurements were collected on days when the cooling treatments were activated. For each measurement date, 9–10 fruitlets per tree were imaged, representing two canopy positions: sun-exposed fruitlets located on the outer canopy and shaded fruitlets located inside the canopy under leaf cover.

Measurements were collected under the two cooling treatments—sprayers and sprinklers—and compared to a control (no cooling) plot. In the first experimental year (26 June 2022), cooling systems were also activated for several hours to test system performance under standard seasonal weather conditions, serving as a baseline reference day (mean air temperature: 32 ± 3 °C). In the second and third years, the temperatures were measured on hot days during cooling activation—4 May 2023 and 24 April 2024—with a mean air temperature of 43 ± 2 °C.

Fruitlet-temperature distributions for each treatment and canopy position were extracted from the thermal images and averaged for comparison of the three years.

#### 4.2.6. Stomatal Conductance

Stomatal conductance was measured on 9 May 2024—a hot day when the evaporative cooling system was operational—at 1000 h on five young, fully grown leaves that were healthy and fully exposed per tree using a LI-COR LI-600 porometer, Lincoln, NE, USA.

### 4.3. Statistical Analysis

JMP^®^ 14.0.0 software (SAS Institute Inc. Cary, NC, USA) was used to carry out analysis of variance (ANOVA). The Tukey–Kramer test was used to estimate the differences between the treatments (SWP; stomatal conductance; Na, Cl, and Ca in leaves; and yield).

The significance of the temperature differences was evaluated through statistical analysis of the average foliage temperatures across different treatment groups. This study determined the statistical significance of the results using *p*-values. For temperature differences between cooled and uncooled trees, a significance threshold of *p* < 0.01 was used. Differences between specific emitter flow rates were also tested with significance levels of *p* < 0.01 and *p* < 0.05.

Statistical analysis was performed to compare the mean fruitlet temperatures among treatments (control, sprayers, and sprinklers), separately for each year, and between canopy positions (sun vs. shade). Treatment means were evaluated using ANOVA and significant differences between treatment groups were determined at *p* < 0.05.

## 5. Conclusions

Heatwave damage to the avocado is prevalent in many of its growing areas due to, for example, the Santa Ana winds in California, the Sharav in Israel, the Berg winds in South Africa, and hot and dry winds in Peru and Australia. Evaporative cooling systems were found to be a highly effective strategy for mitigating the detrimental impacts of heatwaves on ‘Hass’ avocado production. Utilizing a low-discharge rate of 1.5 mm/h, which is significantly lower than the rates used in a previous study, the grower can achieve substantial physiological benefits without overtaxing the hydraulic capacity of commercial irrigation systems.

## Data Availability

The original contributions presented in this study are included in the article/Supplementary Material. Further inquiries can be directed to the corresponding author.

## Supplementary Materials

The following supporting information can be downloaded at https://www.mdpi.com/article/10.3390/plants15101516/s1, Table S1. Operation timing of the evaporative cooling system in the experimental plot.
