# Supplementary material for: Evaluating Overhead Sprinklers and Sprayers for Heatwave Protection in Avocado Orchards
Source: Plants (Basel). 2026 May 15;15(10):1516. doi: 10.3390/plants15101516 (PMC13210729; doi:10.3390/plants15101516)
Supplement: Supplementary file 1 [file plants-15-01516-s001.zip › plants-4250472-supplementary.pdf]

Table S1. Operation timing of the evaporative cooling system in the experimental plot.

Year	Date (day/month)	Time (h)	Operating period (h)	Total operation time (per year)
2022	17/4	1230–1800	5.5	
	3/5	1000–1250	2.8	
	14/5	1110–1615	5.1	
	11/6	1230–1630	4.0	
	26/6	1000–1300	3.0	20.4
2023	19/4	1200–1450	2.8	
	4/5	1000–1430	4.5	
	5/5	0940–1227	2.8	
	1/6	0940–1338	4.0	
	2/6	0949–1901	8.9	
	9/6	1100–1900	8.0	
	17/6	0900–1300	4.0	35.0
2024	24/4	1000–1850	8.8	
	25/4	0839–1736	9.0	
	9/5	0903–1715	8.1	
	27/5	1110–1342	2.5	28.4
